# Sleep Pattern Interference in the Cognitive Performance of Lusitano Horses

**DOI:** 10.3390/ani14020334

**Published:** 2024-01-21

**Authors:** Ângela P. Barbosa, Tiago M. Oliveira, Pedro Henrique E. Trindade, Sarah R. T. Seidel, Paula K. A. Tokawa, Fernando M. Jaramilo, Neimar V. Roncati, Raquel Y. A. Baccarin

**Affiliations:** 1 Faculdade de Medicina Veterinária e Zootecnia (FMVZ), Universidade De São Paulo (USP), São Paulo 05508-270, SP, Brazilbaccarin@usp.br (R.Y.A.B.); 2 Faculdade de Medicina Veterinária e Zootecnia (FMVZ), Universidade Estadual Paulista “Julio De Mesquita Filho” (UNESP), Botucatu 18618-687, SP, Brazil; 3 Grupo Unieduk, Jaguariuna 13918-110, SP, Brazil

**Keywords:** REM sleep, welfare, sleep–wake cycle, learning

## Abstract

**Simple Summary:**

Little is known about how incomplete sleep affects a horse’s memorization. Like most mammals, horses need sleep to fully function, with differences in the total sleep time needed. The literature shows that horses need about three to four hours divided between two phases: the REM (rapid eye movement) phase and the NREM (non-rapid eye movement) phase. The aim of this study was to better understand how interference with the REM phase can affect the memory skill of horses. For this study, 10 horses were filmed in their stall to characterize their sleep patterns for 48 h. Afterwards, horses that experienced REM sleep interference and horses that did not experience REM sleep interference (control) over 72 h were evaluated by performing a spatial memory test and a visual attention test. A time period of 48 h seemed long enough to characterize the individual sleep patterns of the horses. REM sleep deprivation appeared to increase the response time of the horses in spatial memory tasks. This study shows the importance of preserving sleep patterns in horses, and, with further studies, the results could be used to improve welfare conditions for horses.

**Abstract:**

Like most mammalian, polyphasic sleep, equine sleep can be divided into two phases: the REM (rapid eye movement) phase and the NREM (non-rapid eye movement) phase. For this study, a randomized crossover experiment was conducted using ten purebred Lusitano horses, all dressage athletes aged from three to seven years old. The horses were filmed before the intervention to characterize their sleep patterns. REM sleep deprivation was achieved by not letting the horses attain sternal or lateral recumbency for three consecutive days, totaling 72 h. A spatial memory task and a visual attention test were performed. A recording time of 48 h appeared to be long enough to characterize the sleep patterns of the stalled horses. The total recumbency time of the studied population was lower than that previously reported in horses. Although the recumbency times before and after the intervention were similar, there was a tendency shown by the delta (*p* = 0.0839) towards an increased time needed to resolve spatial memory tasks in the sleep-deprived group. Future studies may deepen the understanding of horse sleep requirements and patterns, and the effects of environmental changes on horse sleep.

## 1. Introduction

Sleep is a physiological and behavioral necessity of a diverse range of animals, including mammals. Many physiological regulations occur during this process, which are most prominent in the nervous, cardiovascular, and endocrine systems [1,2,3,4]. Behavioral changes can be observed as well, and can be used, with or without physiological alterations, to assess sleep [4]. The sleep patterns of humans have been very well explored in the literature, and adequate rest is considered one of the pillars of success in therapeutic approaches and as part of training for athletes to achieve victories [5,6]. This theme is rarely mentioned in horses; however, it has been gaining prominence in more recent publications [7,8,9,10]. As with polyphasic sleep in many mammals, equine sleep can be divided into two phases: the REM (rapid eye movement) phase, or paradoxical phase, where rapid eye movement, atony, and the loss of muscle tone occur, and the NREM (non-rapid eye movement) phase [11]. Horses are known to have an adapted sleep pattern that is divided into small fragments [10]. Complete sleep is achieved through cyclical alternation between these phases [12] for around three hours, occurring mainly during the night [10,13]. There are about thirty periods of NREM sleep lasting about three to four minutes throughout the day, and in this case, the animal can rest while standing [13]. However, to reach REM sleep, the animal must be in a decubitus position. This phase is also short and takes from three to five minutes to complete [10,13,14]. Studies on the cognitive capacity of horses are still scarce in comparison with other species, and mainly regard discriminative learning [15]. Although horses have the capacity for discriminative learning, there seem to be limits to this, such as the inability to apply what has been learned to solve new problems [15]. The experimental designs of studies on equine cognitive capacity need to consider factors related to the intrinsic behaviors of this species, such as prey behavior and their ability to read facial expressions, as well as individual factors, such as sex, age, and animal experience [15,16]. Dressage horses must be able to distinguish between highly precise cues from their rider while performing sophisticated movements, so attention during training and competitions is essential [17]. This type of training may be related to lower generalization learning, as this strict training conditions the horse, allowing minimal freedom. Dressage horses also show greater emotional responses than other athlete horses [18]. Above all, there is little research that correlates cognitive ability with purposeful interference in REM sleep patterns [7]. Sleep pattern interference in horses can occur for many reasons; some studies have examined the effects of changing the bedding, lights, or sounds in the study environment [3,4], and have evaluated memory consolidation in response to these changes [7]. Hospitalization can also change sleep patterns as well as disease severity [19], showing that horses are less likely to stay in recumbency as often as normal when they are faced with a new environment. Athlete horses are often exposed to situations of REM sleep deprivation, such as during transport or while sleeping in new environments during competitions. Thus, the objectives of this experiment were to evaluate the sleep patterns of dressage horses, determine how a period of REM sleep deprivation impacts the cognitive skills of horses, and examine the effects of sleep deprivation on subsequent sleep patterns.

## 2. Materials and Methods

### 2.1. Animals

**Figure 1:**
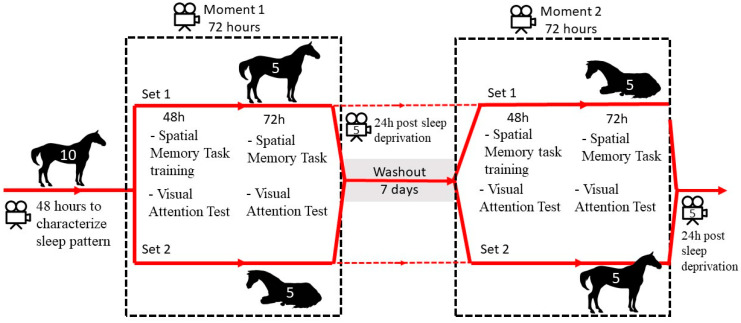
Characterization of the experiment’s timeline.

Ten purebred Lusitano horses were used in this randomized crossover experiment; they were dressage athletes aged from 3 to 7 years old, comprising nine males and one female, and were housed in 4 × 4 m stalls with wood shavings as bedding, an automatic water trough, and two separated troughs for feed and mineral salt. The horses received feed three times a day, with three portions of hay right before each feed. The feeding times were 6 am, 12 pm, and 5 pm. During the experiment, the same husbandry to which the animals were habituated was maintained, meaning that all the horses worked for at least one hour daily, alternating between mounted work in a covered arena and unmounted exercises in a round pen. After working while either mounted or unmounted, the horses received a bath before returning to their stall. The horses did not leave the stalls for anything else. All the animals were housed at the same property for at least a year and were in those respective stalls for two weeks prior to the experimentation. The stalls had small apertures with grids on lateral sides for the horses to see each other. All the animals were recorded in order to characterize their individual sleep pattern in their habitual stalls for 48 h seven days before the interventions began. For logistic reasons, the animals were divided into two sets, with five in each. All ten horses went through two distinct moments: with REM sleep deprivation and without sleep interference. After a washout period of seven days, the interventions were inverted. The first set of five animals to be sleep deprived was chosen at random. Each moment had a duration of 72 h (three days), with one set involving REM sleep deprivation, and the other set receiving no interference. The cognitive performance of the horses was evaluated for each moment using a memory test as well as a visual attention test. All the horses were in their designated stalls for 10 days before the experiment began. The routine of the animals was not altered for the experiment. They did not leave their stalls aside from training, which occurred once a day, every day, for about two hours. The stalls were cleaned twice a day, and the horses were fed three times a day. This work was approved by the Ethics Committee on Animal Use of the School of Veterinary Medicine and Animal Science (University of São Paulo) (CEUA/FMVZ), with protocol number CEUA 6584300721. A characterization of the experiment is visualized in Figure 1.

### 2.2. Cameras

Fixed IP cameras were installed in each of the ten stalls. The cameras had a high image resolution and infrared, enabling the cameras to record during both day and night. The horses were recorded for 24 h during the experiment, including during the washout period. The camera system was coupled to a DVR with a 1Tb HD, with four channels, that recorded 10 days of video. To record ten stalls simultaneously, three DVRs were used. Video backups were made immediately, with an external HD of 2 Tb, using a USB plug. REM sleep deprivation was accomplished by not letting the horses enter into sternal or lateral recumbency for three consecutive days, totaling 72 h. An observer was always looking at the animals, during both the day and night, and would gently stimulate the horses to stand if they laid down. Three observers took turns, staying with the horses for eight hours at a time, until the completion of the 72 h. The animals could roll over in the bedding if they wanted. The REM sleep deprivation started at 11 am on the first day of each moment. The horses stayed in their habitual stalls, without being tied, with food and water ad libitum, and without movement restrictions, except for recumbency. 

### 2.3. Spatial Memory Task

The spatial memory task used was based on the Morris aquatic labyrinth, adapted for equines by [7], which was centered on giving the horses clues to find objects. Four 50-L buckets, one blue and three gray, were equally placed in the round pen. For each animal, the feed was placed in a specific random location, with a random bucket color. Each animal underwent a pre-training period, in which all the buckets were presented. The horse could smell and eat from all the buckets while being led by an observer. After that, in the training phase, each horse was led to the middle of the round pen and set free, and they were given a limited time of 120 s to localize their assigned bucket. The training phase occurred at the 48 h mark of the experiment. The learning criterion was set at four consecutive successful attempts. The training would stop if the horse showed no interest in interacting with the buckets for three consecutive tries. After four consecutive failures, even if the horse interacted with the buckets, the training phase would cease. The test occurred at the 72 h mark. The horses were brought individually to the round pen in the same manner as during the training session and had 120 s to find the correct bucket with feed, as presented in the training phase. Each horse was given four attempts. The time each animal took to complete or fail the test was measured, as well as the number of successful attempts. As the location was important and not the color of the bucket, half of the animals worked with the blue bucket, while the other half worked with the gray bucket, with the colors of the bucket being inverted according to the moments (without deprivation and with sleep deprivation).

### 2.4. Visual Attention Test

The visual attention test was adapted from [20] using a green LED light. The visual stimulus was projected on the back wall of the stall for the horse, always in a circular clockwise movement, intercalating with vertical and horizontal movements in a cross shape, for 60 s. For every animal, the observer would enter the stall, put the halter on, and direct the animal to the back wall of the stall to start the test. Then, the horse was free to move as it wanted. Every session was recorded by a second observer standing close to the stall door. The test was performed in silence to minimize distractions in the form of external sounds. The test was performed after 48 and 72 h of the experimental treatment. It analyzed the reaction time; the attention level, assessed according to the duration of each gaze and the total gaze time; the attention focus, assessed according to the number of gazes over 60 s; and, lastly, the number of times their attention was fragmented (gaze number over 60 s).

### 2.5. Statistical Analysis

All the statistical analyses were performed in the R software with the RStudio integrated development environment (version 4.1.0 (29 June 2021), RStudio, Inc., Boston, MA, USA). The functions and packages used were presented in the format “pacote::função”, corresponding to the programming language in R. For all the tests, a level of 5% was considered significant. To be inclusive, all the figures were built with a color palette distinguishable by colorblind people (ggplot2::scale_colour_viridis_d). A priori, the delta was calculated by subtracting the duration of the training stage from that of the test stage of the memory test when the horse was deprived of sleep and when it was not. A posteriori, the delta, the duration, and the frequency of correct answers in the training and test stages during the memory test, as well as the parameters evaluated in the attention test, were compared between the moments when the horses slept (control) or did not sleep (sleep deprivation) using a paired Student’s *t*-test (stats::t.test) for the data that presented a normal distribution according to the Cramer–von Mises test (nortest::cvm.test), and a paired Wilcoxon test (stats::wilcox.test) when normality was not detected. The number of undefeated horses in the training and test stages during the memory test was compared between moments using a chi-squared test (stats::chisq.test). The sternal and lateral recumbency counts between time points (“basal” vs. “sleep-deprivation” vs. “control” vs. “sleep-pattern-after”) and sets (“first-control” vs. “first-sleep-deprivation”) were conducted with a multilevel generalized linear model adjusted by a Poisson distribution (lme4::glmer). The days and interactions between moments and groups were used as fixed effects, and the horses were considered random effects. Multiple comparisons were conducted post hoc using the Bonferroni test (lsmeans::lsmeans and multcomp::cld). The duration of sternal and lateral recumbency did not show a normal distribution according to the Cramer–von Mises test; therefore, the comparisons between the moments were carried out using the Friedman test (stats::friedman.test and PMCMRplus::frdAllPairsNemenyiTest), and the comparisons between groups using the Wilcoxon test (stats::wilcox.test).

## 3. Results

### 3.1. Sternal and Lateral Recumbency

**Figure 2:**
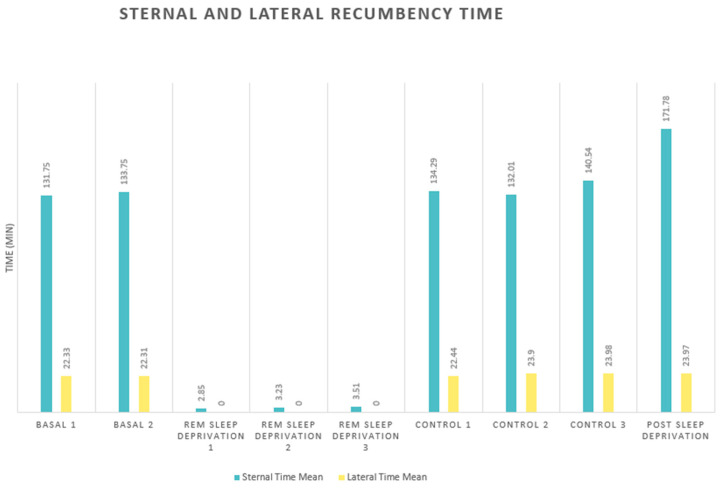
Sternal and lateral recumbency mean times (*n* = 10): basal (days one and two), during sleep deprivation (days one, two, and three), control (days one, two, and three), and post sleep deprivation. Means were measured in minutes.

The mean times, measured in minutes, for sternal and lateral recumbency before the experiment (basal) were 132 and 22, respectively. Looking at the horses that were able to sleep (control), the means were 136 and 23, with means post sleep deprivation of 172 and 24, respectively (Figure 2).

**Figure 3:**
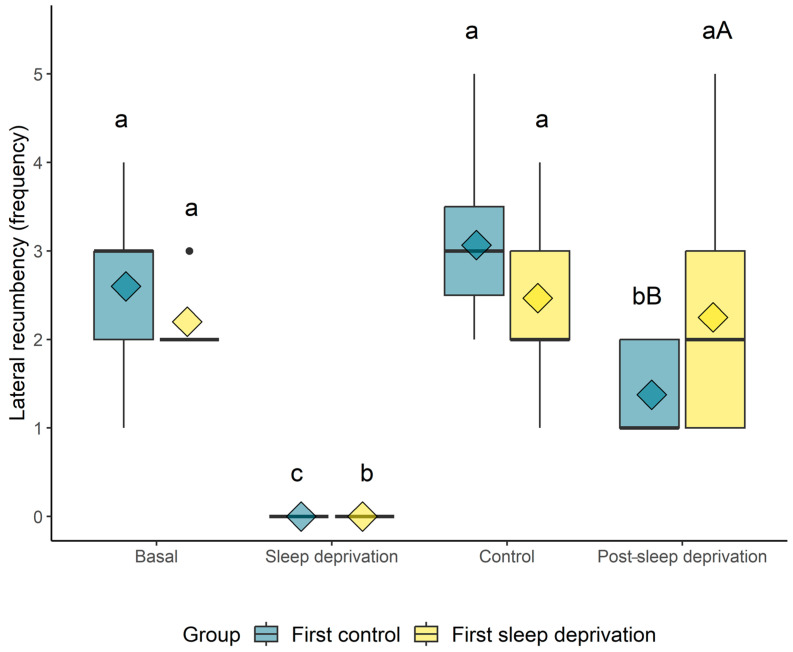
Box plot of the lateral decubitus count when the horses did not sleep (REM sleep deprivation) or slept (control). The diamond indicates the mean; black circles indicate outliers; different lowercase letters indicate statistical differences over time for the same group (a > b > c); and capital letters indicate statistical differences between groups at the same time point (A > B).

The number of lateral recumbency events was statistically equivalent between sets and moments, and there was no influence of day (*p* = 0.9416). In addition, the number of sternal recumbency events had no influence on the day (*p* = 0.9390). However, when the animals were analyzed in separate groups (first-control and first-sleep-deprivation), differences were noticed in the frequency of lateral recumbency between the moments. For both groups (first-control and first-sleep-deprivation), the lateral recumbency count was lower in the “sleep-deprivation” moment compared to the others. In the “first-control” group, the lateral recumbency count was lower in the “post-sleep-deprivation” group compared to the basal (sleep-pattern-before) and “control” moments, while in the “first-sleep-deprivation” group, it was equal (Figure 3). When comparing the groups, the animals first submitted to REM sleep deprivation showed a higher lateral decubitus count at the “sleep-pattern-after” moment in relation to those horses first submitted to the control. 

**Figure 4:**
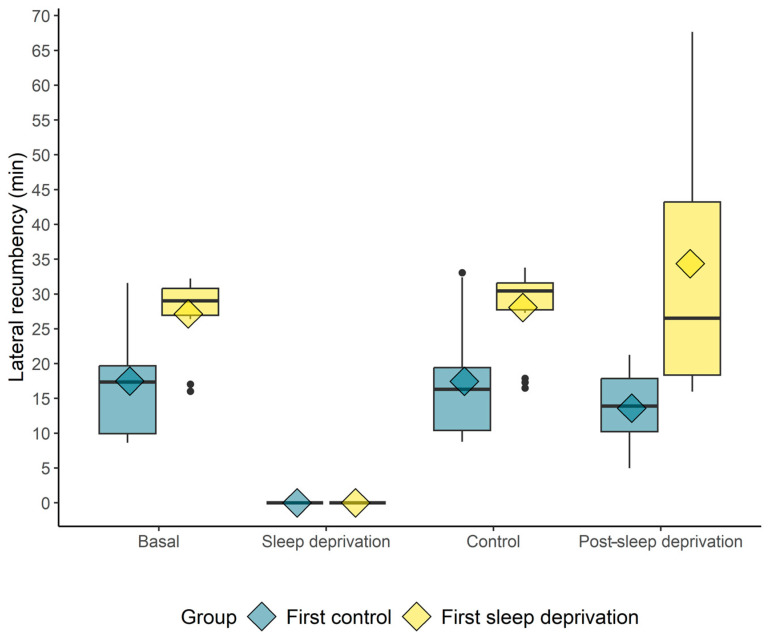
Box plot of the duration (min) of lateral recumbency when the horses did not sleep (REM sleep deprivation) or slept (control). The diamond indicates the mean; black circles indicate outliers.

Regarding the duration of lateral and sternal recumbency, the lateral recumbent time was longer in the basal moment (sleep-pattern-before) only compared to the “sleep deprivation” moment for both groups (Figure 4). When comparing the groups, the animals first submitted to REM sleep deprivation showed a higher lateral recumbency duration in relation to those horses first submitted to the control at all moments except the sleep-deprivation moment (Figure 4).

**Figure 5:**
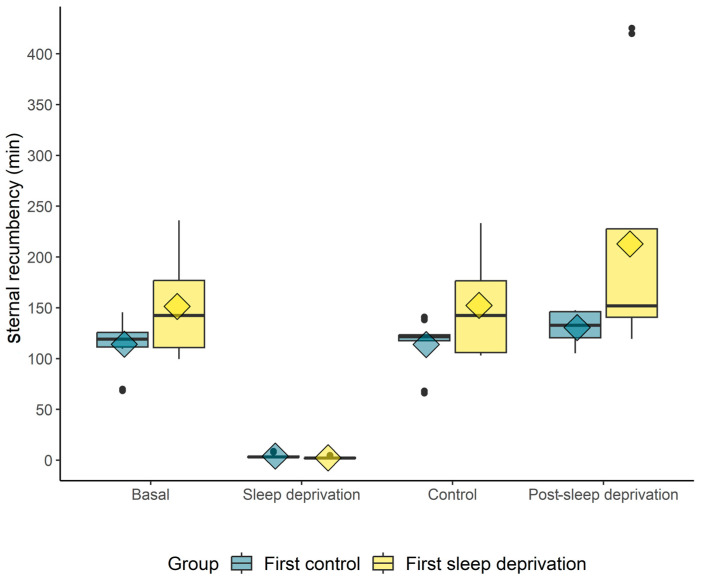
Box plot of the duration (min) of sternal recumbency when the horses did not sleep (REM sleep deprivation) or slept (control). The diamond indicates the mean; black circles indicate outliers.

The sternal recumbency time was longer in the “post-sleep-deprivation” moment than in the “sleep-deprivation” moment for both groups. When comparing the groups, the sternal recumbency duration was greater in the “first-sleep-deprivation” group at the time of sleep deprivation (Figure 5).

### 3.2. Spatial Memory Task

**Figure 6:**
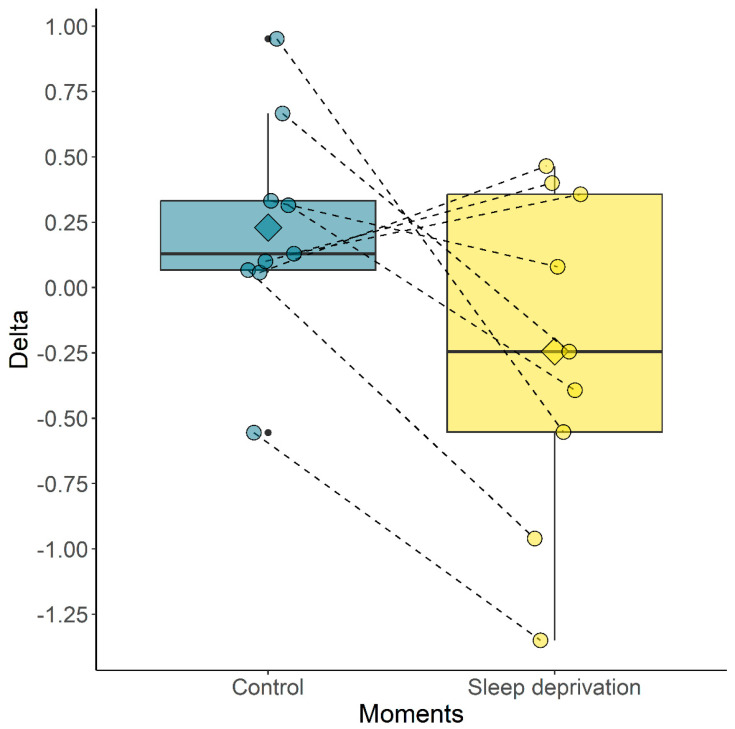
Delta box plot control (horses slept) or sleep deprivation. Diamond indicates mean; black circles indicate outliers; colored circles indicate each horse; dashed lines indicate change in delta between time points.

The delta showed a difference (*p* = 0.0839) between the group where the horses were able to sleep (control) and the group with sleep deprivation (Figure 6). The duration and the frequency of correct answers for the training and test stages during the memory test were statistically equivalent between the control and sleep deprivation groups (Table 1). In the special memory task, only nine horses participated. One of the horses had to be excluded due to acute lameness. The count of undefeated horses in the training and test during the spatial memory task between the control and sleep deprivation groups is shown in Table 2. The training stage gave similar results for both the control and sleep-deprivation groups.

### 3.3. Visual Attention Test

During the attention test, the reaction time at 48 h tended to be greater (*p* = 0.0786) than that of the control group (4.10) for sleep-deprived horses (1.40) (Table 3). The other parameters were statistically equivalent between the control and sleep deprivation groups.

## 4. Discussion

Regarding the sternal (average: 132 min) and lateral (average: 22 min) recumbency time of the studied population, it was observed that the total time was lower than that previously reported in horses: 3 to 4 h [7], 3 h [13], and 3 to 5 h [14]. It is also possible that purebred Lusitano horses have a racial characteristic of less sleep time compared to other breeds, or that this population specifically, for environmental conformities, has a lower amount of required sleep time. This is the first publication characterizing recumbency time in this breed. The total sternal and lateral times for these horses were similar to those found in horses submitted to invasive polysomnographic investigations, with a sternal recumbency time of 111.9 h on average at night, and a lateral recumbency time of 20.2 h at night [15].

This, perhaps, indicates that this population is less susceptible to experiencing changes due to sleep deprivation during short experiments, because they are used to less total sleep. This, evidently, follows individual characteristics, as well as previous present alterations, such as lameness. One of the horses that presented with forelimb lameness before the first moment had an increased sternal recumbency time, in line with the results reported by other studies [19]. The most popular animal models used for examining the effect of REM sleep deprivation or total sleep deprivation are rats and mice, with studies showing that sleep deprivation for 96 h or 192 h can increase the oxidation stress on the liver and pancreas in rats [21], and that it impairs memory, affecting discriminative avoidance in mice [22] and rats [23] in more recent studies. Older studies, as summarized by Rechtschaffen [24], show deleterious effects of prolonged sleep deprivations, such as death, skin lesions, and a debilitated appearance, if total sleep deprivation is forced for more than 96 h. Another popular animal model for sleep deprivation is cats, with similar results as those for rats and mice [25,26,27]. Compared to horses, these three species have a higher total sleep time, which may be the reason why a more visible memory impairment could not be observed after a total of 72 h of REM sleep deprivation in this study.

The horses did not exhibit a significant increase in recumbency time after the period of sleep deprivation in our study, in contrast to that observed in rats [28]. One possible cause could be continued tension, as they were in the same environment where the intervention took place, much like the tension caused in hospitalized horses [19]. This was different from the situation observed in rats, which were removed from their experimental cage and given a recovery period in a different environment [28,29]. The sternal recumbency time was longer in the “post-sleep-deprivation” moment in relation to the “sleep-deprivation” moment for both groups, showing that the interference indeed worked.

The 10 horses were divided into two sets (with 5 in each) for logistical reasons, and they had different basal sleep means. In this regard, it is important to note that being in the first group or second sleep-deprived group had some influence, as the lateral recumbency duration was longer in the “first-sleep-deprivation” group in all cases except when compared with the “sleep-deprivation” group.

Lastly, our results show that the control group’s sleep time was equal to their sleep pattern before the intervention (basal), showing that, in each moment, only the horses in the sleep deprivation groups were sleep deprived. For this group, two nights were necessary to characterize the horses’ sleep patterns, showing that 48 h is the minimum time needed to observe how a horse sleeps without environmental changes.

The correlation between cognition and spatial location has been studied in several species [28]. The spatial memory test was first developed for rats and adapted for other species [29], including equines [7]. Repetitive and standardized tests seem to favor equine learning [29,30], and this characteristic may explain the better performance in the second moment of the experiment, since the horses had already gone through two moments with the same situation, training, and testing as the first moment. In this aspect, it is important to note that both training situations occurred when some horses had already been REM sleep deprived for 48 h; but, according to our data, this factor may not have had any influence overall, as the duration and the correct answers for the training and test stages during the memory test were statistically equivalent between the control and sleep deprivation moments.

Another consideration is regarding the husbandry of the horses. They were not used to being released in the round pen, so many used the moment for other activities, such as rolling or galloping, instead of performing the test. It is worth considering that another kind of attraction should possibly have been used to arouse interest rather than the usual feed. Another factor to consider is the tendency of some horses to present a more cautious approach to new situations, presenting a pessimistic bias [31]. The memory test had similar results to those found by Greening [7], who showed that experimental horses took longer to complete the training phase of the memory test as opposed to the testing phase under treatment conditions. Here, in this study, the delta showed a tendency for horses to take longer to complete the memory test when sleep deprived, but they performed the same in the training phase as the control group. Both studies suggest that there seems to be better memory consolidation when we allow sleep without interference. Sleep has also been reported to improve memory consolidation and the ability to learn new communication skills in dogs, as summarized in the literature [32], with poor sleep being related to emotional and behavioral problems [33]. The visual attention test adapted from Rochais [20] seems to be efficient as an ethological form of assessment, as it allows the expression of different behaviors instead of fixing attention and causing changes in behavior, such as the popular 5-choice serial reaction time test (5-CSRTT), originally developed for rats [34]. Horses’ attention is not only based on their gaze, but also on the position of their ears, so fragmented attention can be observed [35,36].

All horses saw the light signal, with the difference being that some chose to ignore it and interact with the evaluator, and others chose to move away. The animals were also interested in focusing their attention on the evaluator’s hand, which contained the pen that emitted the light signal, instead of the signal itself; thus, it would be interesting to mark the different times spent giving attention to the light signal and to the hand of the evaluator. This behavior seems to demonstrate an attempt to understand human signals [37]. During this test, the reaction time after 48 h tended to be greater (*p* = 0.0786) in the horses that slept (4.10) compared to those that did not sleep (1.40); this difference can perhaps be explained by a more startled behavior from those who did not sleep, with these horses gaining focus more rapidly in new events than those who slept. The horses did not show excessive drowsiness during the 72 h of the experiment, as opposed to the findings reported by other studies [38].

## 5. Conclusions

Different populations can have different sleep patterns. In our study, a group of dressage horses spent less time in recumbency than other horses. When studying stalled horse sleep patterns, 48 h was long enough to record times and characterize them. REM sleep is imperative for horses, and its deprivation appears to cause cognitive impairment, resulting in damage to memory consolidation and attention capacity. Future studies may deepen our understanding of horse sleep requirements and patterns, and how environmental changes affect horse sleep.

## Figures and Tables

**Table 1:** Mean and standard deviation of the parameters evaluated in the special memory task in minutes between control (horses able to sleep) and REM sleep deprivation.

Variable	Control	Sleep Deprivation	Test	*p*-Value
**Memory—training (s)**	0.82 ± 0.67	0.71 ± 0.48	Student’s *t*	0.6512
**Memory—test (s)**	0.59 ± 0.53	0.96 ± 0.71	Wilcoxon	0.1641
**Correct answers—training**	3.11 ± 1.45	3.33 ± 0.86	Wilcoxon	0.7835
**Correct answers—test**	2.77 ± 1.71	2.66 ± 1.58	Wilcoxon	0.9319
**Delta**	0.22 ± 0.42	−0.24 ± 0.63	Student’s *t*	0.0839

**Table 2:** Count of undefeated horses in training and test during the memory test when the horses slept (control) or did not sleep (sleep deprivation).

**Training**		**Undefeated**	
		Yes	No
**Moments**	Control	6	3
	Sleep deprivation	5	4
**Test**		**Undefeated**	
		Yes	No
**Moments**	Control	5	4
	Sleep deprivation	4	5

X-squared = 0, df = 1, *p*-value = 1.

**Table 3:** Mean and standard deviation of the parameters evaluated in the attention test when the horses slept (control) or did not sleep (sleep deprivation).

**48 h**				
**Variable**	**Control**	**Sleep-deprived**	**Test**	***p*-value**
**Reaction time (s)**	4.10 ± 5.03	1.40 ± 0.32	Wilcoxon	0.0786
**Average level of attention (s)**	17.50 ± 13.18	13.50 ± 5.16	Wilcoxon	0.7039
**Total attention time (s)**	37.00 ± 14.88	37.80 ± 13.21	Wilcoxon	0.9395
**Gaze number**	2.70 ± 1.10	2.60 ± 1.02	Wilcoxon	0.9045
**Gaze number index (s)**	0.04 ± 0.02	0.04 ± 0.02	Wilcoxon	0.9045
**72 h**				
**Variable**	**Control**	**Sleep-deprived**	**Test**	***p*-value**
**Reaction time (s)**	2.40 ± 2.73	3.20 ± 4.79	Wilcoxon	0.8360
**Average level of attention (s)**	19.40 ± 15.23	17.50 ± 16.48	Wilcoxon	0.5963
**Total attention time (s)**	34.20 ± 17.67	34.00 ± 19.25	Wilcoxon	0.8795
**Gaze number**	2.00 ± 0.89	1.90 ± 1.30	Wilcoxon	0.6923
**Gaze number index (s)**	0.03 ± 0.01	0.03 ± 0.02	Wilcoxon	0.6923

## Data Availability

The data presented in this study are available from the corresponding author upon request. The data are not publicly available due to the privacy requested by the animals’ owners, as the horses are high-performance animals.
